# Effect of Immediate Referral vs a Brief Problem-solving Intervention for Screen-Detected Peripartum Depression: A Randomized Clinical Trial

**DOI:** 10.1001/jamanetworkopen.2023.13151

**Published:** 2023-05-12

**Authors:** Mei Elansary, Caroline J. Kistin, Jocelyn Antonio, Ivys Fernández-Pastrana, Aviva Lee-Parritz, Howard Cabral, Emily S. Miller, Michael Silverstein

**Affiliations:** 1 Division of General Pediatrics, Department of Pediatrics, Boston University School of Medicine, Boston Medical Center, Boston, Massachusetts; 2 Brown University School of Public Health and Hassenfeld Child Health Innovation Institute, Providence, Rhode Island; 3 Department of Obstetrics and Gynecology, Boston University School of Medicine, Boston Medical Center, Boston, Massachusetts; 4 Department of Biostatistics, Boston University School of Public Health, Boston, Massachusetts; 5 Department of Obstetrics and Gynecology, Division of Maternal Fetal Medicine, Warren Alpert Medical School of Brown University, Providence, Rhode Island

## Abstract

**Importance:**

The US Preventive Services Task Force recommends screening adults for depression in settings with programs in place to ensure receipt of appropriate care. Best practices regarding how to ensure such care are unknown, particularly for pregnant and postpartum persons.

**Objective:**

To compare the effectiveness of 2 strategies for the initial management of screen-detected peripartum depressive symptoms.

**Design, Setting, and Participants:**

This randomized comparative effectiveness trial was performed from February 1, 2018, to June 30, 2020, at the prenatal clinic, postpartum unit, and pediatric clinic within an urban safety-net hospital. Participants included peripartum persons with positive depression screen results. Data were analyzed from July 6, 2020, to September 21, 2022, based on intention to treat.

**Interventions:**

Engagement-focused care coordination (EFCC), which used shared decision-making and motivational techniques to refer patients to outside mental health services, and problem-solving education (PSE), a brief cognitive-behavioral program delivered at the screening site.

**Main Outcomes and Measures:**

The primary outcome consisted of severity of depressive symptoms; secondary outcomes included severity of anxiety symptoms and engagement with care, each measured bimonthly over 12 months. Rates of symptom elevations were modeled using negative binomial regression; rates of symptom trajectories were modeled using treatment × time interactions.

**Results:**

Among the 230 participants (mean [SD] age, 29.8 [5.8] years), 125 (54.3%) were Black and 101 (43.9%) were Hispanic or Latina. At baseline, 117 participants (50.9%) reported at least moderately severe depressive symptoms (Quick Inventory of Depressive Symptomatology score ≥11), and 56 (24.3%) reported clinically significant anxiety symptoms (Beck Anxiety Inventory score ≥21). Across 6 assessment time points, the mean (SD) number of moderately severe depressive symptom episodes in EFCC was 2.2 (2.2), compared with 2.2 (2.1) in PSE, for an adjusted rate ratio (aRR) of 0.95 (95% CI, 0.77-1.17). The mean (SD) number of anxiety symptom elevations in EFCC was 1.1 (1.8), compared to 1.1 (1.6) in PSE, for an aRR of 0.98 (95% CI, 0.69-1.39). There were significant treatment × time interactions relative to mean depressive symptom scores (−0.34 [95% CI, −0.60 to −0.08]; *P* = .009 for interaction term), favoring EFCC. There were no differences in engagement with care.

**Conclusions and Relevance:**

In this randomized comparative effectiveness trial, there were no differences in depressive or anxiety symptom burden across comparators; however, the evidence suggested improved depressive symptom trajectory with immediate referral. Further work is necessary to guide approaches to management following depression screening for peripartum persons.

**Trial Registration:**

ClinicalTrials.gov Identifier: NCT03221556

## Key Points

**Question:**

Is an immediate, activated referral to care or a brief problem-solving intervention more effective for the initial management of screen-detected depression in pregnant and postpartum persons?

**Findings:**

In this randomized clinical trial of 230 pregnant and postpartum participants followed up for 12 months, there were no significant differences in the rate of depressive or anxiety symptom episodes between groups; however, the evidence suggested improved symptom trajectory following immediate referral.

**Meaning:**

Further work is necessary to guide approaches to ensuring adequate follow-up care following a positive depression screen result among peripartum persons.

## Teaser

This randomized clinical trial compares the effectiveness of immediate referral strategy with short-term management of symptoms among pregnant and peripartum persons with screen-detected depression.

## Introduction

In 2016, the US Preventive Services Task Force (USPSTF) updated its recommendation to screen adults for depression in primary care,^1^ and it explicitly applied that recommendation to pregnant and postpartum persons.^2^ The USPSTF asserted that screening for depression confers benefits only when supports exist to ensure that patients with screen-positive findings are properly diagnosed and treated or referred to settings that can provide appropriate care.^1^ Defining optimal postscreening supports, therefore, is critical to maximizing the benefits of depression screening.

A range of approaches have been deployed to provide these supports: staff to facilitate referrals, nurse specialists for formal assessment, brief intervention and referral, or team-based collaborative care.^3,4,5,6^ Although each of these approaches has evidence for improving outcomes, 1 fundamental difference among them is the degree to which each initiates depression management in the primary care setting or refers to outside venues. This distinction is particularly salient for peripartum populations, who historically lack access to evidence-based mental health care. Few obstetric primary care venues, for example, have resources to implement collaborative care,^7^ many obstetric clinicians are ill equipped to treat depression without specialist support,^8^ and many postpartum patients are lost to follow-up.^9^ Consequently, only a minority of patients with perinatal depression receive mental health care,^10^ and fewer are treated to remission.^11^ These problems are compounded for low-income patients and patients from marginalized communities by additional barriers to care and a disproportionate incidence of behavioral comorbidities that make specialty care necessary.^10,12,13,14,15^

We sought to compare the effectiveness of 2 approaches to the initial management of depression among a screen-detected population of pregnant and postpartum persons. We compared immediate referral using an engagement-focused care coordination (EFCC) strategy with short-term management of symptoms using a manualized cognitive behavioral program called problem-solving education (PSE). Whereas EFCC provides referral to formal mental health services outside primary care, PSE offers a brief, on-site, evidence-based treatment, followed by referral to further treatment if symptoms persist. While both EFCC^16,17,18^ and PSE^19,20,21,22^ have been tested among diverse populations and parents of young children,^23,24,25,26,27,28^ they have not been directly compared following universal depression screening.

## Methods

### Design and Setting

We conducted a randomized, pragmatic,^29^ parallel group, comparative effectiveness trial. The trial protocol is provided in Supplement 1. The study was conducted in an urban safety-net hospital in Boston, Massachusetts. We recruited from the prenatal clinic, postpartum unit, and pediatric primary care clinic. The Boston University Medical Center Institutional Review Board approved this study. All participants provided written informed consent. We followed the Consolidated Standards of Reporting Trials (CONSORT) reporting guideline.

### Participants

We recruited pregnant and postpartum persons with depressive symptoms according to the Edinburgh Postnatal Depression Scale, a screening instrument valid during pregnancy and post partum.^30^ To maximize sensitivity and specificity, we defined a positive screen result as a score of 10 or greater.^31^ We selected broad inclusion criteria, excluding only those with suicidal ideation, psychosis, active substance use, or cognitive limitation, according to the MacArthur Competence Assessment Tool.^32^ Participants had to have no current source of mental health care, defined as no more than 1 mental health care appointment in the last 3 months or no scheduled future appointments. They had to be pregnant with the intent to parent their newborn or have delivered a child 0 to 18 months of age at the time of recruitment, be 18 years or older, be conversant in English or Spanish, and live within a prespecified distance from the medical center.

### Recruitment and Enrollment

We enrolled participants from February 1, 2018, to June 30, 2019. Where possible, recruitment procedures harnessed existing screening activities at each recruitment site. In the prenatal clinic, front desk staff conducted screenings at all prenatal visits; in pediatrics, practice assistants completed screenings at all well-child visits. The postpartum unit was added as a recruitment venue 3 months into the enrollment period to address an initial slow rate of study enrollment; because depression screening was not routinely performed in that setting, research staff directly performed the screenings. Clinicians were alerted to all positive screens. Patients were informed directly of their result and option to participate in the study. Among interested patients, research staff assessed eligibility and obtained informed consent.

### Randomization

We used stratified, blocked 1:1 randomization at the participant level. Investigators and data collectors were blinded to study allocation. Randomization occurred independently at each recruitment site, stratified by whether participants had previously received depression treatment, in blocks of randomly varying sizes of 2 and 4 within strata. To conceal randomization, study staff accessed a secure webpage to allocate study groups.

### Study Groups

Engagement-focused care coordination emphasized referral to depression services following a brief engagement session; PSE offered initial on-site treatment, followed by referral if depressive symptoms persisted. Patients in both groups had access to the same array of mental health services. Treatments in both groups were delivered by bachelor’s level clinical staff with no prior mental health care experience. Intervention training lasted 2 days for EFCC and 5 days for PSE and included confirmatory fidelity checks. To minimize contamination, we divided staff into those trained in EFCC and those trained in PSE. During the study, participants were assigned to the language-concordant, study group–specific clinical staff member with the smallest caseload at the time.

#### Engagement-Focused Care Coordination

Based on frameworks of motivational and ethnographic interviewing, EFCC followed the core components of the evidence-based engagement interview^16^: clinical staff disclosed the probable diagnosis of depression, provided psychoeducation, presented treatment options, and engaged clients in shared decision-making to determine the most appropriate referral. Patients in the EFCC group received 1 or 2 sessions delivered by telephone.

#### Problem-solving Education

Problem-solving education is an evidence based-depression care model that includes 6 one-on-one, workbook-based problem-solving sessions, depressive symptom monitoring, and linkage to mental health services for participants with refractory or worsening symptoms.^25,33^ Problem-solving sessions lasted 30 to 45 minutes and were delivered weekly or biweekly, primarily through home visitation. Sessions focused on defining a problem, goal setting, and generating and implementing solutions. Participants with moderate symptoms on 3 successive assessments, or anyone who developed severe symptoms, were referred to mental health services.

### Baseline Data

Prior to randomization, participants self-reported their age and number of children, race (from a list that included Asian, Black, White, and other or >1 race), ethnicity (Hispanic or Latina), educational level, work status, single- vs dual-parent family, and receipt of public assistance programs. We assessed depressive symptoms with the Quick Inventory of Depressive Symptomology (QIDS), using the cut points of 11 or greater as the threshold for moderately severe symptoms and 14 or greater for severe symptoms.^34^ Depression history was assessed with the Mini-International Neuropsychiatric Interview, version 6.0.^35^ We assessed anxiety symptoms with the Beck Anxiety Inventory, using a cut point of 21 or greater to define clinically significant symptoms.^36^ Trauma history and posttraumatic stress disorder (PTSD) symptoms were assessed using the Modified PTSD Symptom Scale.^37^ Alcohol dependence was assessed using the Alcohol Use Disorders Identification Test^38^; perceived stress, using the Perceived Stress Scale.^39,40^

### Outcome Assessment

We followed up participants at 2-month intervals for a total of 12 months post randomization, ending June 30, 2020. The primary outcome was the rate of depressive symptom episodes, operationalized as elevations to the moderately severe threshold; we conducted sensitivity analyses using the severe symptom threshold. Anxiety symptoms, a secondary outcome, were assessed concurrently.

Additional outcomes included engagement and retention with mental health services, measured by the National Institute of Mental Health’s Collaborative Psychiatric Epidemiology Studies.^41^ Consistent with prior literature,^10^ we defined engagement as at least 1 visit for psychotherapy or pharmacotherapy with a social worker, psychologist, psychiatrist, or psychiatric nurse or a prescription for psychiatric medication by a general medical or obstetrical clinician. We defined retention in care as 4 or more visits, and evidence-based care as 4 or more specialty or general health care visits over 12 months plus use of an antidepressant medication or 8 or more specialty visits in the absence of antidepressant use.^42^

### Statistical Analysis

Data were analyzed from July 6, 2020, to September 21, 2022. We conducted our analyses using SAS, version 9.4 (SAS Institute Inc). Intention-to-treat analyses compared differences in outcomes across treatment groups. For depression and anxiety, we conducted direct comparisons of symptom episodes at each time point and examined symptom trajectories using treatment × time interactions. We used negative binomial regression to compare the incident rates of symptomatic episodes over 12 months of follow-up. Incident rates were calculated as the number of times an individual exceeded a symptom threshold divided by the number of assessments; we used an offset to standardize rates according to the number of assessments completed. An episode was defined as any follow-up time point at which a study participant scored over a designated threshold. Consistent with prior work,^24,25,43^ we adjusted all models assessing depressive symptoms for baseline QIDS scores (and models assessing anxiety symptoms for baseline Beck Anxiety Inventory scores) to estimate adjusted rate ratios (aRRs).

To examine symptom trajectories, we used generalized estimating equations to examine group × time effects on mean depressive and anxiety symptom scores. Consistent with prior work,^25^ we estimated a primary model that excluded baseline symptoms in the set outcomes but adjusted for them. To ensure the results were stable across plausible models, we also estimated an unadjusted model that included baseline symptoms in the set of outcomes and an unadjusted model that excluded baseline symptoms in the set of outcomes.

To assess engagement and retention in care, we conducted direct comparisons at each time point. We conducted serial cross-sectional comparisons in which engagement or retention at each time point was considered independently. We also examined engagement as a cumulative phenomenon, such that anyone engaged with care at a proximal time point was considered engaged thereafter. We used logistic regression to compare the proportion of participants who met the definition of engagement, retention, or receipt of evidence-based care over the full follow-up period and adjusted models for whether participants had engaged with mental health care in the 6 months prior to enrollment.

**Figure 1.**
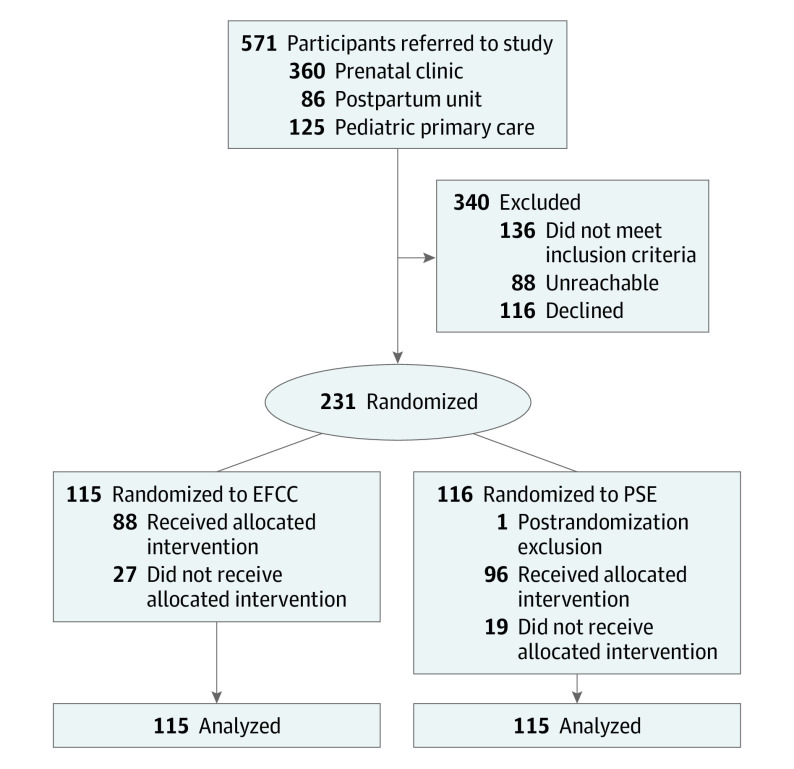
Study Flow Diagram EFCC indicates engagement-focused care coordination; PSE, problem-solving education.

We examined heterogeneity of treatment effect according to whether participants were recruited during pregnancy or post partum and according to whether participants had a history of trauma. Because of our low rate of missing data overall and at each follow-up time point (Figure 1), we did not impute missing data.

We estimated our sample size to provide power to test a clinically significant difference across intervention groups on rate of symptom elevations. We estimated that a sample size of 100 per group would be able to detect a clinically important 33% reduction in the rate of symptom elevations from 1.2 to 0.8 per 6 follow-up assessments in each group in a Poisson model. Our sample size of 230 assumed 80% power, a 2-sided α = .05 indicating statistical significance, and 15% loss to follow-up.

## Results

### Enrollment

In total, 571 individuals were referred to the study (Figure 1): 360 from the prenatal clinic, 86 from the postpartum service, and 125 from the pediatric primary care clinic (where parents brought their infants for care). Across all referrals, 136 (23.8%) were ineligible, 39 for having a current source of mental health care, 39 for not being conversant in English or Spanish, 20 for substance use, 17 for not being pregnant or for having a child younger than 18 months, 15 for living outside the geographic range of the study, 3 for cognitive limitation, and 1 each for suicidality, planning to terminate the pregnancy, and planning to place their newborn for adoption. Among the remaining 435 individuals, 204 (46.9%) were unreachable or declined participation (eTable 1 in Supplement 2). There was a single postrandomization exclusion of a participant in the PSE group who had a miscarriage after being randomized, leaving 230 trial participants.

### Baseline Characteristics

Among the 230 participants, 3 (1.3%) identified as Asian; 125 (54.3%), as Black; 30 (13.0%), as White; and 50 (21.7%), as other (responses included Cape Verdean, complicated, don’t know, Hispanic, Hondurian, Indigenous, Latina, Latino, Mestiza, none, Puerto Rican, and West Indian) or more than 1 race. One hundred and one participants (43.9%) identified as Hispanic or Latina (Table 1). The mean (SD) age was 29.8 (5.8) years. One hundred seventeen participants (50.9%) reported at least moderately severe depressive symptoms; 59 (25.7%), severe depressive symptoms; 56 (24.3%), anxiety symptoms; and 136 (59.1%), a lifetime traumatic event. Seventy participants (30.4%) met symptom criteria for PTSD. Most baseline characteristics were balanced across comparators; exceptions included race (68 [59.1%] in EFCC identified as Black vs 57 [49.6%] in PSE), ethnicity (42 [36.5%] in EFCC identified as Hispanic or Latina vs 59 [51.3%] in PSE), and working outside the home (60 [52.2%] in EFCC vs 44 [38.3%] in PSE).

**Table 1.** Baseline Characteristics of Participants

Characteristic	Participant group^a^		
	Overall (n = 230)	Treatment	
		EFCC (n = 115)	PSE (n = 115)
Maternal demographic characteristics			
Age, mean (SD), y	29.8 (5.8)	29.6 (5.8)	30.1 (5.9)
Race			
Asian	3 (1.3)	2 (1.7)	1 (0.9)
Black	125 (54.3)	68 (59.1)	57 (49.6)
White	30 (13.0)	9 (7.8)	21 (18.3)
Other or >1 race^b^	50 (21.7)	23 (20.0)	27 (23.5)
Missing	22 (9.6)	13 (11.3)	9 (7.8)
Hispanic or Latina ethnicity	101 (43.9)	42 (36.5)	59 (51.3)
Home language			
English	164 (71.3)	81 (70.4)	83 (72.2)
Spanish	92 (40.0)	39 (33.9)	53 (46.1)
Other^c^	46 (20.0)	32 (27.8)	14 (12.2)
Born in the US	102 (43.9)	54 (47.0)	48 (41.7)
Educational level			
Less than high school, including GED	68 (29.6)	26 (22.6)	42 (36.5)
Some college	65 (28.3)	41 (35.7)	24 (20.9)
College degree or higher	51 (22.2)	27 (23.5)	24 (20.9)
Works outside of the home	104 (45.2)	60 (52.2)	44 (38.3)
Single-parent household	126 (54.8)	66 (57.4)	60 (52.2)
No. in household, mean (SD)	5 (2)	5 (3)	4 (2)
No. of children, mean (SD)	2 (1)	2 (1)	2 (1)
Current unstable housing	70 (30.4)	36 (31.3)	34 (29.6)
Family receives TANF, SSI, WIC, or SNAP	172 (74.8)	90 (78.3)	82 (71.3)
Maternal mental health measures			
QIDS depressive symptom score, mean (SD)^d^	11 (5)	11 (5)	11 (5)
QIDS score ≥11	117 (50.9)	56 (48.7)	61 (53.0)
QIDS score ≥14	59 (25.7)	28 (24.3)	31 (27.0)
History of depression^e^	62 (27.0)	34 (29.6)	28 (24.3)
Current anxiety^f^	56 (24.3)	29 (25.2)	27 (23.5)
Trauma history^g^	136 (59.1)	66 (57.4)	70 (60.9)
PTSD^h^	70 (30.4)	36 (31.3)	34 (29.6)
Alcohol dependence or some dependence^i^	16 (7.0)	8 (7.0)	8 (7.0)
Perceived Stress Scale score, mean (SD)^j^	29 (8)	29 (8)	29 (8)
Engagement with care^k^			
Engaged with care	121 (52.6)	60 (52.2)	61 (53.0)
Took a psychiatric medicine	6 (2.6)	4 (3.5)	2 (1.7)
Saw a behavioral specialist	121 (52.6)	60 (52.2)	61 (53.0)
Enrollment site			
Prenatal clinic	101 (43.9)	50 (43.5)	51 (44.3)
Pediatrics clinic	67 (29.1)	34 (29.6)	33 (28.7)
Postpartum unit	62 (27.0)	31 (27.0)	31 (27.0)

Abbreviations: EFCC, engagement-focused care coordination; GED, General Educational Development; PSE, problem-solving education; PTSD, posttraumatic stress disorder; QIDS, Quick Inventory of Depressive Symptoms; SNAP, Supplemental Nutrition Assistance Program; SSI, Supplemental Security Income; TANF, Temporary Assistance for Needy Families; WIC, Women, Infants, and Children program.

^a^ Unless otherwise indicated, data are expressed as No. (%) of participants.

^b^ Includes participants identifying as any other race not listed; responses provided by participants included Cape Verdean, complicated, don’t know, Hispanic, Hondurian, Indigenous, Latina, Latino, Mestiza, none, Puerto Rican, and West Indian.

^c^ Participants reported another language as the one most commonly spoken at home and also spoke either English or Spanish.

^d^ Scores range from 0 to 27; 11 or greater indicates moderately severe depressive symptoms, and 14 or greater indicates more severe depressive symptoms.

^e^ Measured using the Mini-International Neuropsychiatric Interview.

^f^ Indicates Beck Anxiety Inventory score of 21 or greater.

^g^ Identified using the Modified PTSD Symptom Scale, Stem Question.

^h^ Identified using the Modified PTSD Symptom Scale, Symptom Thresholds.

^i^ Measured using the Alcohol Use Disorders Identification Test.

^j^ Possible scores range from 0 to 40, with 0 indicating low perceived stress and 40 indicating high perceived stress.

^k^ Services section of the Collaborative Psychiatric Epidemiology Studies.

### Intervention Delivery, Fidelity, and Safety

Across 9 EFCC clinical staff, caseloads ranged from 5 to 26 participants; across 4 PSE clinical staff, caseloads ranged from 16 to 41 participants. Engagement-focused care coordination was designed to be 1 or 2 sessions in length: 28 participants (24.3%) received 1 session; 60 (52.2%) received 2 sessions; and 27 (23.5%) did not receive any sessions. Problem-solving education was designed to be 6 sessions in length: 52 participants (45.2%) received at least 4 sessions; 27 (23.5%) completed 6 sessions; and 19 (16.5%) did not receive any sessions. Thirty-one participants in the PSE group were referred to further mental health care. No adverse events occurred.

### Depressive Symptoms

Although the proportion of participants with moderately severe depressive symptoms decreased from baseline in each group, on average participants spent greater than one-third of the follow-up period with moderately severe symptoms; and there were no differences across comparators (Table 2). The mean (SD) number of moderately severe depression symptom elevations in EFCC was 2.2 (2.2), compared with 2.2 (2.1) in PSE (aRR, 0.95 [95% CI, 0.77-1.17]). In a sensitivity analysis of severe depressive symptoms, the mean (SD) number of episodes in EFCC was 1.2 (1.6) compared with 1.3 (1.7) in PSE (aRR, 0.84 [95% CI, 0.63-1.12]).

**Table 2.** Proportion of Participants Meeting Symptom Thresholds for Moderate Depression and Anxiety by Intervention Comparator^a^

	Participant group					
	Depressive symptom episode			Anxiety symptom episode		
	EFCC	PSE	*P* value^b^	EFCC	PSE	*P* value^b^
Baseline (n = 230)	56/115 (48.7)	61/115 (53.0)	.51	29/115 (25.2)	27/115 (23.5)	.76
Follow-up time point						
2 mo (n = 223)	32/113 (28.3)	26/110 (23.6)	.43	14/113 (12.4)	11/110 (10.0)	.57
4 mo (n = 221)	29/112 (25.9)	29/109 (26.6)	.90	15/112 (13.4)	12/109 (11.0)	.59
6 mo (n = 202)	33/105 (31.4)	29/97 (29.9)	.81	18/105 (17.1)	17/97 (17.5)	.94
8 mo (n = 216)	34/109 (31.2)	33/107 (30.8)	.96	18/109 (16.5)	15/107 (14.0)	.61
10 mo (n = 221)	30/112 (26.8)	32/109 (29.4)	.67	16/112 (14.3)	19/109 (17.4)	.52
12 mo (n = 220)	34/112 (30.4)	44/108 (40.7)	.11	22/112 (19.6)	24/108 (22.2)	.64
No. of symptomatic episodes, mean (SD)	2.2 (2.2)	2.2 (2.1)	NA	1.1 (1.8)	1.1 (1.6)	NA
aIRR (95% CI)^c^	0.95 (0.77-1.17)	1 [Reference]	NA	0.98 (0.69-1.39)	1 [Reference]	NA

Abbreviations: aIRR, adjusted incident rate ratio; EFCC, engagement-focused care coordination; PSE, problem-solving education; NA, not applicable.

^a^ Unless indicated otherwise, data are presented as the No./total No. (%) of participants.

^b^ Reflects χ^2^ test at each cross-sectional time point.

^c^ Adjusted for baseline symptoms.

**Figure 2.**
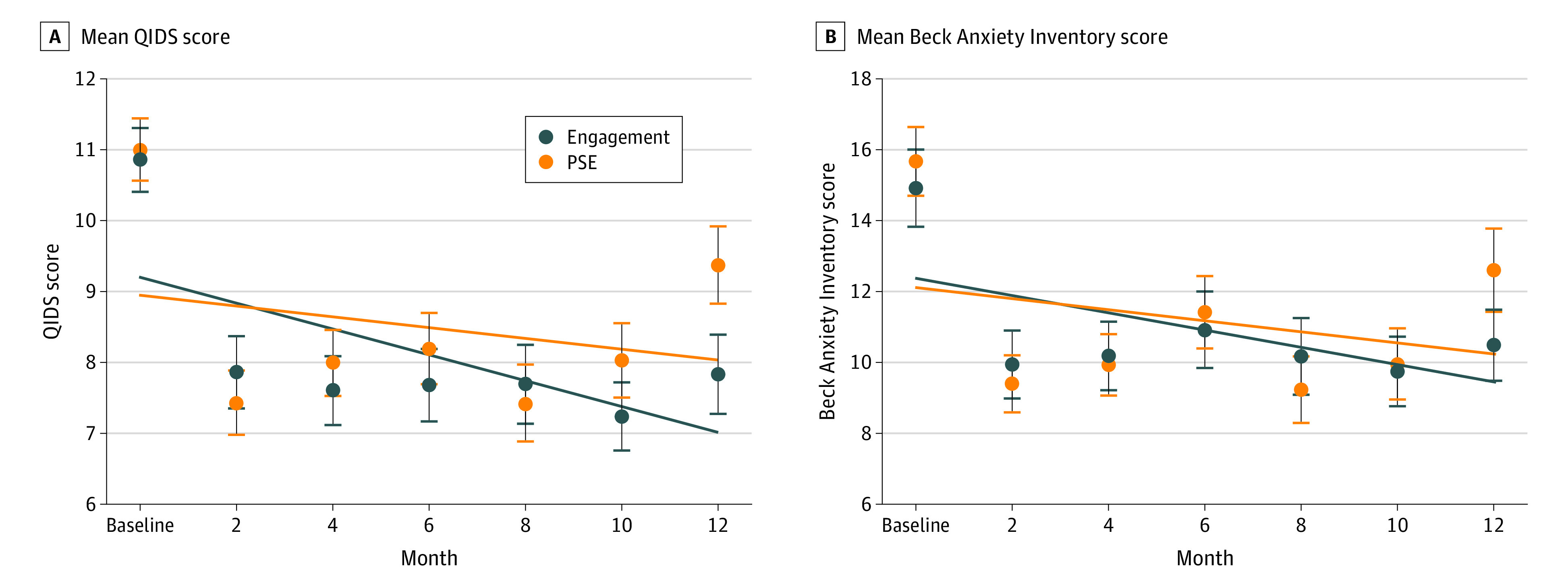
Trajectory of Depressive and Anxiety Symptoms Over Time A, Depressive symptom trajectories were assessed using a treatment × time interaction term. Mean depressive symptom scores were assessed using the Quick Inventory of Depressive Symptomology (QIDS) with a group × time interaction term. Model excluded baseline depression symptoms in the set outcomes but adjusted for them. Dots indicate mean QIDS score; vertical lines and whiskers indicate 95% CIs. B, Anxiety symptom trajectories were assessed using treatment × time interaction term. Mean anxiety symptom scores were assessed using the Beck Anxiety Inventory with a group × time interaction term adjusted for baseline score. Model excluded baseline anxiety symptoms in the set outcomes but adjusted for them. Dots indicate mean Beck Anxiety Inventory score; vertical lines and whiskers indicate 95% CIs.

Across study groups, there were significant differences in depressive symptom trajectories (Figure 2). Although mean QIDS scores remained similar between groups from baseline through 10 months, there was a significant difference at the 12-month time point, with a mean (SD) score of 7.8 (5.9) in EFCC compared with 9.4 (5.7) in PSE (*P* = .05). This difference appeared to drive a statistically significant treatment × time interaction that demonstrated a difference in the trajectory of depressive symptoms over the follow-up period. Specifically, in a regression model adjusting for baseline depressive symptoms, a treatment × time interaction (−0.34 [95% CI, −0.60 to −0.08]; *P* = .009) suggested a symptom trajectory favoring EFCC. These results were consistent across alternative models (eTable 2 in Supplement 2).

### Anxiety Symptoms

The proportion of participants with anxiety symptoms decreased from baseline in each group during the follow-up period (Table 2). However, there were no significant differences across comparators at each time point. The mean (SD) number of anxiety symptom elevations in EFCC was 1.1 (1.8), compared with 1.1 (1.6) in PSE (aRR, 0.98 [95% CI, 0.69-1.39]). In a model adjusting for baseline anxiety symptoms, a treatment × time interaction (−0.52 [95% CI, −1.04 to 0.001]; *P* = .05) suggested a symptom trajectory favoring EFCC (Figure 2). This finding was inconsistent across alternative models (eTable 2 in Supplement 2).

### Engagement and Retention in Care

There were no differences in engagement with care across groups at each cross-sectional time point nor in cumulative engagement over time (Table 3). Of those not engaged with care at baseline, 42 participants (36.5%) in EFCC subsequently engaged with mental health care, compared with 37 (32.2%) in PSE (adjusted odds ratio, 1.21 [95% CI, 0.69-2.14]). Twenty-one persons (18.3%) in EFCC were retained in mental health care, compared with 20 (17.4%) in PSE (adjusted odds ratio, 1.06 [95% CI, 0.53-2.11]).

**Table 3.** Participants' Engagement With Care Over the Follow-up Period^a^

Time point	Participant group											
	Engaged with care				Took a psychiatric medicine				Visited behavioral health specialist			
	Cross-sectional analysis		Cumulative analysis		Cross-sectional analysis		Cumulative analysis		Cross-sectional analysis		Cumulative analysis	
	EFCC	PSE	EFCC	PSE	EFCC	PSE	EFCC	PSE	EFCC	PSE	EFCC	PSE
2 mo	15/113 (13.3)	13/110 (11.8)	15/113 (13.3)	13/110 (11.8)	6/113 (5.3)	8/110 (7.3)	6/113 (5.3)	8/110 (7.3)	12/113 (10.6)	10/110 (9.1)	12/113 (10.6)	10/110 (9.1)
4 mo	19/112 (17.0)	22/109 (20.2)	23/112 (20.5)	25/109 (22.9)	9/112 (8.0)	13/109 (11.9)	10/112 (8.9)	14/109 (12.8)	11/112 (9.8)	16/109 (14.7)	17/112 (15.2)	21/109 (19.3)
6 mo	17/105 (16.2)	22/97 (22.7)	25/105 (23.8)	27/97 (27.8)	8/105 (7.6)	10/97 (10.3)	11/105 (10.5)	14/97 (14.4)	13/105 (12.4)	17/97 (17.5)	21/105 (20.0)	24/97 (24.7)
8 mo	17/109 (15.6)	20/107 (18.7)	31/109 (28.4)	32/107 (29.9)	10/109 (9.2)	12/107 (11.2)	13/109 (11.9)	16/107 (15.0)	10/109 (9.2)	12/107 (11.2)	27/109 (24.8)	27/107 (25.2)
10 mo	21/112 (18.8)	24/109 (22.0)	37/112 (33.0)	36/109 (33.0)	13/112 (11.6)	14/109 (12.8)	16/112 (14.3)	18/109 (16.5)	11/112 (9.8)	12/109 (11.0)	33/112 (29.5)	30/109 (27.5)
12 mo	22/112 (19.6)	26/108 (24.1)	45/112 (40.2)	42/108 (38.9)	11/112 (9.8)	14/108 (13.0)	19/112 (17.0)	21/108 (19.4)	15/112 (13.4)	17/108 (15.7)	41/112 (36.6)	36/108 (33.3)

Abbreviations: EFCC, engagement-focused care coordination; PSE, problem-solving education.

^a^ Data are presented as No./total No. (%) of participants. The cross-sectional analysis considers each time point independently; the cumulative analysis carries forward prior engagement to subsequent time points.

### Heterogeneity of Treatment Effect

There was no evidence of heterogeneity of treatment effect in stratified analyses according to enrollment during or after pregnancy (eTable 3 in Supplement 2). The interaction term for trauma history on the relationship between comparator and outcomes was not significant (eTable 4 in Supplement 2).

## Discussion

We compared 2 strategies for the initial management of screen-detected depression among pregnant and postpartum patients. The study was designed to address an evidence gap in the USPSTF’s recommendation for depression screening. The comparison between EFCC and PSE answered the question whether providing peer-delivered cognitive behavioral therapy as an initial, postscreening depression treatment improved outcomes compared with coordinated referral to mental health services.

Our results demonstrated that participants experienced at least moderately severe depression symptoms over one-third of the 12-month follow-up period. However, the rate of symptomatic person-time—for both depression and anxiety—was nearly identical across comparators. Toward the end of the follow-up period, depressive symptom scores appeared greater among those receiving PSE. This difference drove a statistically significant treatment × time interaction that demonstrated a difference in the trajectory of depressive symptoms favoring EFCC. We also detected significant treatment × time interactions for anxiety symptoms in a subset of models. These differential symptom trajectories, which we interpret as clearly present for depression but more equivocal for anxiety, did not appear to result from differential engagement with formal mental health care across comparators.

### Strengths and Limitations

The main strength of our study is its randomized design. Our diverse sample—with more than one-half of participants identifying as Black and nearly one-half identifying as Hispanic or Latina—makes our contribution to that literature important in light of the USPSTF’s recent call for diversifying the evidence base for preventive interventions to populations underrepresented in research.^44^ Our study, however, has limitations. The first is suboptimal uptake of both interventions. While this phenomenon is expected in the context of an effectiveness study, it appeared more severe in the EFCC group and is compounded by each intervention group having an intentional different number of contacts. The second limitation is our lack of ability to explain the underlying mechanisms for differential symptom trajectories across the study groups. While conducting a study within a single medical center minimizes potential biases associated with heterogeneity in local mental health resources, it also poses limitations to generalizability. Last, while our comparative effectiveness design allowed for direct comparison of EFCC and PSE, the absence of a usual care group precluded the ability to definitively state that either were superior compared with usual care.

## Conclusions

The USPSTF has determined that depression screening confers meaningful benefits only when supports are in place to ensure that patients are appropriately diagnosed and treated or referred to a setting that can provide appropriate care.^1^ While our study confirmed both the burden of depression in the peripartum period and the challenges of engaging non–treatment-seeking individuals with depression in care, it was unable to demonstrate the superiority of one approach over another in responding to a positive depression screen result. However, there was suggestive evidence of improved depressive trajectories with immediate referral. This line of research is critical to the provision of quality care for peripartum persons, particularly those from low-income and minority backgrounds. Further work to determine the optimal course of action following perinatal depression screening is critical. In the absence of robust systems of team-based mental health care for pregnant and postpartum persons, using evidence-based techniques to refer patients directly to mental health care following a positive depression screen result appears equally as effective as initiating preliminary on-site treatment.

## Notes

Supplement 1.
Trial Protocol

Supplement 2. **eTable 1.** Excluded Participants, by Recruitment Venue **eTable 2.** Depression and Anxiety Symptom Trajectories **eTable 3.** Incident Rates of Episodes of Clinically Significant Depression and Anxiety Symptoms, by Recruitment Venue **eTable 4.** Experience of Trauma on the Relationship Between Comparator and Outcomes

Supplement 3.
Data Sharing Statement
