# Supplementary material for: Effect of Immediate Referral vs a Brief Problem-solving Intervention for Screen-Detected Peripartum Depression: A Randomized Clinical Trial
Source: JAMA Netw Open. 2023 May 12;6(5):e2313151. doi: 10.1001/jamanetworkopen.2023.13151 (PMC10182435; doi:10.1001/jamanetworkopen.2023.13151)
Supplement: Supplement 1. — Trial Protocol [file jamanetwopen-e2313151-s001.pdf]

PROTOCOL TITLE

ClinicalTrials.gov number: NCT03221556
Protocol Version Number: 1.3
Protocol Version Date: December 12, 2019
Funding Mechanism: Patient Centered Outcomes Research Institute, contract #AD-1603-34662
Principal Investigator: Michael Silverstein, MD MPH
Phone: 617-414-7903
E-mail: Michael.Silverstein@bmc.org

CONFIDENTIAL

This document is confidential and the property of Boston Medical Center / Boston University. No part of it may be transmitted, reproduced, published, or used by other persons without prior written authorization from the study sponsor.

TABLE OF CONTENTS

1 List of Abbreviations ... 3
2 Protocol Summary ... 3
3 Background/Rationale & Purpose ... 4
    3.1 Background Information ... 4
    3.2 Rationale and Purpose ... 5
4 Objectives ... 5
    4.1 Study Objectives ... 5
    4.2 Study Outcome Measures ... 6
        4.2.1 Primary Outcome Measures ... 6
        4.2.2 Secondary Outcome Measures ... 6
5 Study Design ... 7
6 Potential Risks and Benefits ... 7
    6.1 Risks ... 7
    6.2 Potential Benefits ... 8
    6.3 Analysis of Risks in Relation to Benefits ... 8
7 Study Subject Selection ... 8
    7.1 Subject Inclusion and Exclusion Criteria ... 8
8 Study Intervention ... 9
9 Study Procedures ... 9
10 Assessment of Safety and Data Safety Monitoring Plan (DSMP) ... 12
    10.1 Definitions ... 12
    10.2 Safety Review ... 13
    10.3 Reporting Plans ... 14
    10.4 Stopping Rules ... 14
11 Data Handling and Record Keeping ... 14
    11.1 Confidentiality ... 14
    11.2 Source Documents ... 15
    11.3 Case Report Forms ... 15
    11.4 Study Records Retention ... 16
12 Statistical Plan ... 16
    12.1 Study Hypotheses ... 16
    12.2 Sample Size Determination ... 16
    12.3 Statistical Methods ... 17
13 Ethics/Protection of Human Subjects ... 18
14 References ... 19
15 Appendix ... 20

1 List of Abbreviations

Abbreviation	Abbreviation definition
PSE	Problem Solving Education
EPDS	Edinburgh Prenatal Depression Screen
BMC	Boston Medical Center
QIDS	Quick Inventory of Depressive Symptoms
PCMH	Patient Centered Medical Home
USPSTF	United States Preventive Services Task Force

2 Protocol Summary

**Title:**	Improving Outcomes for Low-Income Mothers with Depression: A Comparative Effectiveness Trial of Two Brief Interventions in the Patient-Centered Medical Home (PCMH)
**Population:**	230 English or Spanish-speaking pregnant or postpartum women with depressive symptoms. Pregnant women are patients at BMC's Prenatal Clinic; postpartum women are patients in BMC's postpartum unit, or they have children birth to 18 months who are patients at BMC's primary care pediatrics clinic.
**Interventions:**	Arm #1: Engagement-focused care coordination<br>Arm #2: Problem Solving Education (PSE)
**Objectives:**	1. Effectiveness. Compare the effectiveness of Engagement-Focused Care Coordination and PSE on key patient-reported outcomes, including depression and anxiety symptoms, coping skills, self-efficacy, parenting stress, and child behavior; and on mothers' engagement with formal mental health treatment.<br>2. Heterogeneity of Treatment Effect. Determine whether timing of intervention delivery (pregnancy or postpartum) moderates the difference between the two comparators.<br>3. Implementation. Conduct qualitative interviews with patients, clinic administrators, staff, and intervention providers to understand perspectives and organizational cultures that serve as barriers or facilitators to implementing the comparators in real world practice settings (This implementation aim will be the topic of a separate IRB application).
**Design/Methodology:**	We are conducting a parallel group randomized controlled trial (n=230) to compare the effectiveness of two care coordination models among low-income mothers with depressive symptoms. The first comparator is engagement-focused care management, with its presumed active ingredient, the Engagement Interview. The second comparator is PSE. Over a 12-month follow-up period, we will compare the effectiveness of these two conditions in

	improving depression (primary outcome measure) and anxiety symptoms, parental self-efficacy and coping skills, parenting stress, and child behavior.<br><br>In the outpatient setting, all pregnant women seeking care in BMC's prenatal clinic, and all mothers of young children seeking care in BMC's primary care pediatric clinic, will be screened for depressive symptoms with the EPDS. A positive screen will prompt a member of each clinic's care team to contact the screened patient. This care team member will offer the opportunity to participate in the trial, or to receive usual support provided to women with positive depression screens.<br><br>In the inpatient setting, women who meet study eligibility in BMC's postpartum unit will be approached to be screened for depressive symptoms with the EPDS and for study eligibility with the verbal screen (upon verbal consent). Women with a positive EPDS screen and verbal screen will be offered participation in the trial.<br><br>Those opting to participate in the trial will meet with a study team member for written informed consent and for determination of final eligibility. Study participants must have no current source of mental health care and speak English or Spanish. Following confirmation of eligibility, the study staff member will administer a baseline survey that includes valid and reliable scales for depression and anxiety symptoms, trauma history and post-traumatic stress symptoms, history of receipt of mental health care, co-morbid substance use, and participant demographics. Mothers will then be randomized 1:1 to the two comparators, and followed for 12 calendar months.
**Total Study Duration:**	Three years
**Subject Participation Duration:**	One year

3 Background/Rationale & Purpose

3.1 Background Information

In 2016, the United States Preventive Services Task Force (USPSTF) updated its positive recommendation for screening adults for depression in primary care [1]. For the first time, it designated pregnant and postpartum women as specific groups among whom universal screening can lead to improved outcomes [2]. The USPSTF, however, also cited the caveat that identifying individuals with depression confers meaningful benefits only in settings that have 'adequate systems' in place to assure effective treatment following a positive screen [1]. Despite a higher incidence of maternal depression among low-income and minority populations [3], these mothers face numerous barriers to effective treatment [4, 5] – leading to poor outcomes for both themselves and their children [3]. For this population, determining which medical home-based systems produce the best patient-centered outcomes is critical to reducing mental health disparities.

We are conducting a type 1 hybrid effectiveness-implementation study [6], whereby we concurrently compare two depression care management strategies, and systematically analyze barriers and facilitators to their adoption in the prenatal, pediatric primary care, and post-partum floor settings (Note: the secondary implementation analysis is the subject of another IRB application). Both intervention strategies (also referred to as comparators) are based on the construct of Screening, Brief Intervention, and Referral to Treatment (SBIRT) [7]. While both comparators share the same universal screening strategy, they differ in their brief intervention models, and in when mothers are referred to formal depression care. The first comparator is engagement-focused care management, in which the brief intervention is the Engagement Interview [8, 9]. In this model, bachelor-level care managers meet one to two times with mothers who screen positive for depression, and use techniques of shared decision making and motivation building to help mothers process the results of the screen; explore their related concerns; and connect with depression treatment services. The second comparator is Problem Solving Education (PSE), in which the brief intervention is a six-session cognitive-behavioral model, also delivered by bachelor-level providers [10, 11]. Whereas the Engagement Interview emphasizes referral to culturally relevant depression services, the PSE comparator offers immediate, low-level depression treatment onsite, and is followed by referral to further treatment if depressive symptoms persist. Over 12 months of follow-up, we will track patient-centered outcomes and engagement with care.

This study will be conducted in compliance with the protocol, applicable regulatory requirements, and BMC/BU Medical Campus Human Research Protection policies and procedures.

3.2 Rationale and Purpose

Optimizing care coordination programs within the PCMH for populations with known health disparities is one of the IOM’s top priorities for comparative effectiveness research [12]. Our trial is designed specifically to evaluate whether providing first-step depression treatment in the PCMH – delivered by peer providers – enhances uptake of services and improves outcomes among a screened population of pregnant and postpartum women; or whether it serves to delay more definitive care and symptom relief. Answering this question will allow a screened population of low-income and minority mothers to make informed decisions regarding how and where to receive depression care; and it will allow practice managers to make informed decisions regarding how to organize key components of the PCMH for both pregnant and postpartum women. Our ultimate goal is to reduce disparities in access to mental health care for low-income mothers; and to improve outcomes for them and their children. Our study also addresses a key evidence gap related to the USPSTF’s latest recommendation on depression screening.

4 Objectives

4.1 Study Objectives

Our **study objectives** are as follows:

1. Effectiveness. Compare the effectiveness of Engagement-Focused Care Coordination and PSE on key patient-reported outcomes, including depression and anxiety symptoms, coping skills, self-efficacy, parenting stress, and child behavior; and on mothers’ engagement with formal

mental health treatment.

2. Heterogeneity of Treatment Effect. Determine whether timing of intervention delivery (pregnancy or postpartum) moderates the difference between the two comparators.
3. Implementation. Conduct qualitative interviews with patients, clinic administrators, staff, and intervention providers to understand perspectives and organizational cultures that serve as barriers or facilitators to implementing the comparators in real world practice settings.

4.2 Study Outcome Measures

4.2.1 Primary Outcome Measures

Each subject will have 12-months of follow-up. All outcomes will be assessed at 2-month intervals.

Our primary outcome measure is depressive symptomatology, as measured by the QIDS – and operationalized as an incident rate of moderate to severe episodes over time and as symptom trajectories.

4.2.2 Secondary Outcome Measures

We have identified a series of additional patient-centered outcomes pertaining to symptom relief, behavioral activation, parenting stress, self-efficacy, coping skills, and child behavior.

a. Symptom relief and behavioral activation. Structured surveys and interviews have consistently demonstrated that the most meaningful outcomes to patients involve a combination of symptom relief and functional improvement [13]: symptom relief is more relevant when linked to the feeling of being activated (Spanish word used, 'ánimo'), 'being in a better place,' or feeling like one is actively 'climbing out of the hole.' In addition to the QIDS, we will also administer the Beck Anxiety Inventory every other month will allow us to follow anxiety symptom trajectories. We will accompany these measures with the Behavioral Activation for Depression Scale.

b. Self-efficacy and coping skills. Engagement Interview and PSE participants tend to discuss self-efficacy and coping skills in terms of their ability to manage a stressful event or get through an episode of demoralization. The Brief COPE measures ways of coping with stress through various scales, including (but not limited to): self-distraction, active coping, denial, substance use, and use of emotional support.

c. Quality of Life. We will be using the Individual Burden of Illness Index for Depression, which incorporates multi-dimensional patient-reported outcomes from the QIDS, Quality of Life Enjoyment and Satisfaction Questionnaire - Short Form (Q-LES-Q-SF), and the Work and Social Adjustment Scale (WSAS).

d. Parenting and Perceived Stress. Our parent advisors frequently raise the issue of stress – particularly as it relates to parenting. The Parenting Stress Index – Short Form is one of few valid and reliable tools that can assess a wide range of parenting behaviors in a single instrument, including attachment to child, social isolation, competence, relationship with spouse, and parental health. Cronbach's α for the parent domain is 0.93 and the test-retest coefficient is 0.96 [17]. We will also

use the Perceived Stress Scale (PSS-14), the domains of which include unpredictability, lack of control, burden overload, and stressful circumstances.

e. Child behaviors. Almost uniformly, our patient partners emphasize the impact of child behavior on their daily quality of life. For this, we will use the Child Behavior Checklist (CBCL-1.5/5) to assess a range of internalizing and externalizing child behaviors for children 1.5-5 years. We realize that not all participants will have children in this age range at all assessment time-points; thus, we have intentionally not powered the study with this measure.

An additional secondary outcome is engagement and retention with mental health services, as measured by NIMH's Collaborative Psychiatric Epidemiology Survey [18], which records all primary, specialty, and alternative sources of care. We categorize participants into those who engage with care; those retained in care; and those who receive evidence-based care. We use these definitions because of their association with a high likelihood of full symptom remission [5, 19, 20].

5 Study Design

We are conducting a randomized, parallel group comparative effectiveness trial of 230 pregnant and post-partum women with symptoms of depression. Women in one arm will receive Engagement-Focused Care Coordination, and women in the other will receive Problem Solving Education (PSE). Both intervention arms have been the subject of multiple BUMC IRB applications; all participant safety protocols have been tested over more than 10 years of related research.

In Engagement-Focused Care Coordination, the brief intervention is the Engagement Interview, which is designed to help engage participants in mental health care. The Engagement Interview is NOT a therapeutic or preventive intervention in and of itself. In delivering the Engagement Interview, Bachelor-level providers meet one to two times with women who screen positive for depression, and use techniques of shared decision-making to help mothers process the results of the screen; explore treatment options; and connect with formal mental health services. In PSE, problem solving sessions are one-on-one, workbook-based interactions that typically last 30 to 45 minutes. Sessions are delivered in the home, during lunch breaks from work, or in the clinic. A full course involves 6 sessions, delivered weekly or biweekly.

We will employ stratified, blocked 1:1 randomization, the unit of which will be the mother. All investigators and data collectors will be blinded to study allocation. Randomization will occur independently at each clinic site (Prenatal Clinic, Pediatric Primary Care, Postpartum Unit). We will further stratify randomization by whether or not participants have previously received medication or therapy for depression, as this will likely impact treatment choices. Randomizing in blocks of randomly varying sizes of 2 and 4 within stratum will ensure balance between study arms.

Follow-up data collection will occur over the course of 12 month, with repeated collection of patient-centered outcomes by self-reported to research team members masked to intervention arm.

6 Potential Risks and Benefits

6.1 Risks

Potential risks are as follows:

- Because the research covers the subject of depression, emotionally sensitive subject matter may be discussed. This may be emotionally distressing to individuals in the study.
- Although we will strive to maximize the cultural sensitivity in delivering the proposed interventions, it is possible that certain individuals' explanatory models of their conditions will be incompatible with our proposed interventions. This has the potential to upset study subjects.
- Although we will make every effort to store data in a secure and confidential manner, in concordance with IRB approval, breeches of confidentiality may occur accidentally.
- On rare occasion, information may be obtained that may require mandatory reporting (for example, if a mother divulges that her boyfriend abuses her children). Although we have developed protocols to address such scenarios, they will invariably be upsetting.

6.2 Potential Benefits

The subjects involved in the study stand a reasonable chance of benefiting from both interventions, as both have RCT-level evidence of effectiveness. This study follows from greater than 10 years of prior work documenting its safety when conducted by the current group of investigators. Individuals not directly involved in this work stand the opportunity to benefit from the knowledge we gain from the study.

6.3 Analysis of Risks in Relation to Benefits

The potential long-term benefits of this study plan outweigh its risks. Determining effective therapies and prevention strategies for maternal depression—particularly strategies accessible to low-income populations—represents an opportunity not only to help depressed women, but also to enhance developmental outcomes for their children.

7 Study Subject Selection

7.1 Subject Inclusion and Exclusion Criteria

Inclusion Criteria	Exclusion Criteria
• Woman is pregnant and receives prenatal care at BMC; or is biological mother of 0 to 18-month-old child receiving care at BMC pediatric primary care clinic<br>• Woman has EPDS score ≥ 10 (the most common cutoff used in studies included in the USPSTF report [2])<br>• Woman comfortable speaking and receiving information in English or Spanish<br>• Woman had only one mental health care appointment	• Woman under 18 years of age<br>• Woman endorses suicidality<br>• Woman exhibits signs of psychosis or is cognitively limited [a]

in the last 3 months OR, if more than one appointment, woman has no upcoming appointments.	

8 Study Intervention

We are comparing 2 interventions in this trial: Engagement-Focused Care Coordination and PSE.

In Engagement-Focused Care Coordination, the brief intervention is the Engagement Interview, which is designed to help engage participants in mental health care. The Engagement Interview is NOT a therapeutic or preventive intervention in and of itself. In delivering the Engagement Interview, Bachelor-level providers meet one to two times for 45-60 minutes with women who screen positive for depression, and use techniques of shared decision-making to help mothers process the results of the screen; explore treatment options; and connect with formal mental health services. Engagement Interview training will be based on existing manualized protocols.

In PSE, problem solving sessions are one-on-one, workbook-based interactions that typically last 30 to 45 minutes. Sessions are delivered in the home, during lunch breaks from work, or in the clinic. A full course involves 6 sessions, delivered weekly or biweekly. The PSE training curriculum takes place over the course of a few weeks. After a two-day workshop, in which trainees will receive didactic instruction, they will complete five learning cases that will be audiotaped and reviewed.

9 Study Procedures

Please see the Appendix for the Schedule of Events.

The entire study duration will last from 5/1/2017 to 4/30/2020. However, the duration of subject participation will be 12 months for each subject. Here, we will describe all study procedures, including recruitment, screening, interventions, and follow-up.

<u>Recruitment Procedures</u>

In the outpatient setting, recruitment and enrollment into this trial will be linked to existing clinical systems in BMC's Pediatric Primary Care Clinic (Department of Pediatrics) and BMC's Prenatal Clinic and Postpartum Unit (Department of Obstetrics/Gynecology). Specifically, recruitment procedures will center around existing depression screening activities, common to both clinic sites; and embedded behavioral health services, which are a part of the current standard of care within Prenatal Clinic. Most importantly, all pregnant women seeking care in BMC’s prenatal clinic and all mothers of young children seeking care in BMC’s primary care pediatric clinic are screened with the EPDS - which is the primary instrument that determines eligibility into the PCORI study.

After a woman checks in for a prenatal appointment with Ob/Gyn or for a Pediatric appointment for her child, while waiting to be called into an exam room, she is screened for depression with the EPDS (attached). Upon completing the screen, the mother is informed of her score, its clinical significance, and treatment options (i.e. treatment options that are unrelated to the study). As the principal recruitment mechanism into the study, we will embed a “study option” into this clinical algorithm for all

those with EPDS scores ≥ 10. Specifically, the possibility of learning more about the study will be presented as part of a menu of options to receive behavioral health services. If the mother expresses interest in being contacted for follow-up, she will provide contact information to the clinic staff member to be shared with the study team.

Once alerted that there is a new, EPDS-positive woman who is interested in follow-up, the study team member of the study team will review the mother's medical record for study eligibility, and will contact the mother soon thereafter to explain the study in detail and do further screening. We feel that is it appropriate for the staff member to review limited fields in the EHR (participant's age, child's age, participant's insurance status, address, past and upcoming behavioral health appointments, language, and visit type on the date of the EPDS screen) because this will allow for the determination of appropriate exclusion criteria with the least burden to the potential participant.

Further, if the mother is ineligible for the study after this further screening, the study team member will offer to connect the mother to existing clinic services. If she is eligible for the study, the team member will arrange with the mother a meeting in which consent, final eligibility determination, baseline, and randomization will take place.

In the inpatient setting, recruitment and enrollment will occur on BMC's post-partum floor. The eligibility criteria for this new postpartum population will be the same as our existing participants. The details of our approach are as follows:

1. Low literacy, language-appropriate, written information about the study (provided as an attachment in the application) will be provided to all families in their welcome packet to BMC’s labor and delivery services.
2. Study RAs will confirm with hospital staff that the families have received the packet; once that has been confirmed, RAs will approach families according to the recruitment script attached to the application and entitled "Recruitment Script – Postpartum Packet".
3. Study staff will confirm both the mother's and baby's health status with hospital staff prior to introducing themselves to the mother for potential participation in the study.
4. Approaching mothers will occur in the postpartum unit, in concordance with guidelines from hospital staff. If the infant is hospitalized in the NICU or the inpatient ward, it is possible that will be approaching the mothers in these areas.
5. Consistent with other BMC studies, we will give potential subjects 24 hours after a vaginal delivery as a recruitment time frame and 48 hours after a c-section.
6. In the event of an early discharge, RAs will get permission from hospital staff to call the mother directly by phone. RAs will only call the mother if either the RA had made contact with the mother and explained the study, if the hospital staff member talked to the mother about the study, or if the staff member can confirm that the mother received her welcome packet.
7. Once the RA has made contact with the potential participant, all procedures will mirror what is already written in the study protocol.

Screening Procedures

In the outpatient setting, the first stage of screening will occur as part of existing clinic practice. After a woman checks in with Ob/Gyn or for a Pediatric appointment for her child, she will be screened for depression with the EPDS. If she scores a 10 or above, she will be presented with a menu of treatment options (according to clinic protocols), among which learning more about the study will also be offered as an option. If the woman expresses interest in being contacted for follow-up, clinic staff will ask her to provide contact information to pass along to the study team.

Once her contact information has been shared with the study team, a study team member will perform eligibility screening via the mother's medical record. We will do so in order to minimize unnecessary contact with subjects who are ultimately not eligible, and to minimize the time burden experienced by potential subjects on the phone with the study team. From the mother's medical record, the study team will gather the following: maternal age, child's age (if mother was recruited from the pediatric clinic), maternal health insurance type, maternal address, mother's past and upcoming behavioral health appointments, and the visit type on the date of the EPDS screen. If the mother is ineligible based on the medical record review, the team will connect her to existing clinic services. If the mother is eligible after the record review, the team will explain the study in more detail and ask for verbal consent to administer a few additional screening questions. These screening questions will ask about fluency in English or Spanish, whether the mother is currently receiving behavioral health services, and her pregnancy status. If the mother is ineligible based on any of her responses, the study team will explain that she is not eligible for the study at this time and offer to connect her to existing clinic resources. If the mother is eligible based on her responses, the study team will arrange a meeting in which written informed consent, final eligibility determination, baseline, and randomization will take place.

In the inpatient setting, the first stage of screening will occur when the research staff member reviews the daily census of the postpartum unit. The research staff will review: the mother's age, maternal health insurance, maternal address, mother's phone number, and mother's past and upcoming behavioral health appointments (if available). The second stage of screening will occur when the research staff member approaches the mother per the recruitment protocol. If the mother agrees to be screened (verbal consent), the staff member will administer the EPDS in-person, as well as the verbal eligibility screen. If she scores less than a 10 or scores a 10 or greater but fails the verbal screen, she will be informed that she is not eligible for the study. If she scores a 10 or above and passes the verbal screen, she will be informed that she is eligible for the study and the study team will arrange a meeting in which written informed consent, final eligibility determination, baseline, and randomization will take place.

At the meeting for informed consent, the final stage of screening will take place. After written informed consent, study staff will administer the MacArthur Competence Assessment Tool for Clinical Research (MacCAT-CR) and a suicide screen. For safety reasons, mothers who endorse suicidality at baseline or have disordered thinking will be excluded from the study at this point. Subjects who are eligible after consent will go on to complete the baseline interview and randomization. A safety protocol for managing suicidal ideation is attached to the application.

<u>Intervention Procedures</u>

Following randomization, assigned providers from either intervention arm will reach out to participants to coordinate meetings. Engagement-Focused Care Coordination will entail one to two visits, lasting 45-60 minutes each. PSE will involve six visits, each lasting 30-45 minutes. Meetings for either intervention can take place at home, during a work break, or at the clinic.

<u>Follow-Up Procedures</u>

Following randomization, follow-up assessments will occur every 2 months for 12 months. Follow-up assessors will be blinded to each subject's allocation. The study team will use the preferred methods of contact indicated by the subject upon enrollment (ie. phone calls, texts, email, etc). We will assess certain outcomes measures – depressive symptomatology, anxiety symptom relief, and engagement, retention, and satisfaction with mental health services – at 2 months, 4 months, 8 months, and 10 months by phone. We will assess all outcome measures at 6 months and 12 months in person. Assessment windows will be 6 weeks long: 2 weeks before the "expected date" and 4 weeks after the "expected date." Due to the written consent form having been updated on 4/28/2018, follow-up will also include the reconsenting of participants that were enrolled and consented with the 8/10/2017 consent form.

**Outcome Measure**	**Measured by:**
Depressive symptomatology	QIDS
Symptom relief and behavioral activation	Beck Anxiety Index; Behavioral Activation for Depression Scale
Self-efficacy and coping skills	Brief COPE
Parenting Stress	Parenting Stress Index – Short Form; Perceived Stress Scale
Child behaviors	Child Behavior Checklist (CBCL-1.5/5)
Engagement, retention, and satisfaction with mental health services	Collaborative Psychiatric Epidemiology Survey; Client Satisfaction Questionnaire (CSQ-8)

10 Assessment of Safety and Data Safety Monitoring Plan (DSMP)

10.1 Definitions

The following definitions will be used in the assessment of safety:

Adverse Event (AE) is any untoward or unfavorable medical occurrence in a human subject, including any abnormal sign (for example, abnormal physical exam or laboratory finding), symptom, or disease, temporally associated with the subject's participation in the research, whether or not considered related to the subject's participation in the research.

Serious Adverse Event (SAE) is any adverse event that
(1) results in death;
(2) is life-threatening;
(3) results in inpatient hospitalization or prolongation of existing hospitalization;
(4) results in a persistent or significant disability/incapacity;
(5) results in a congenital anomaly/birth defect; or

(6) based upon appropriate medical judgment, may jeopardize the subject's health and may require medical or surgical intervention to prevent one of the other outcomes listed in this definition (examples of such events include allergic bronchospasm requiring intensive treatment in the emergency room or at home, blood dyscrasias or convulsions that do not result in inpatient hospitalization, or the development of drug dependency or drug abuse).

Life-threatening means that the event places the subject at immediate risk of death from the event as it occurred.

Unanticipated Problem is defined as an event, experience or outcome that meets **all three** of the following criteria:

- <u>is unexpected</u>; AND
- <u>is related or possibly related</u> to participation in the research; AND
- suggests that the research <u>places subjects or others at a greater risk</u> of harm (including physical, psychological, economic, or social harm) than was previously known or recognized.

Possibly related means there is a reasonable possibility that the incident, experience, or outcome may have been caused by the procedures involved in the research

Unexpected means the nature, severity, or frequency of the event is not consistent with either:

- the known or foreseeable risk of adverse events associated with the procedures involved in the research that are described in (a) the protocol–related documents, such as the IRB-approved research protocol, any applicable investigator brochure, and the current IRB-approved informed consent document, and (b) other relevant sources of information, such as product labeling and package inserts; or
- the expected natural progression of any underlying disease, disorder, or condition of the subject(s) experiencing the adverse event and the subject's predisposing risk factor profile for the adverse event.

10.2 Safety Review

Both the risks listed in Section 4.1 and unknown risks will be monitored as follows:

Throughout the course of the study, all procedures and staff conduct will be monitored on an ongoing basis by each site co-investigator (Dr. Lee-Parritz for prenatal clinic and the postpartum floor; Dr. Kistin for pediatric clinic). Data concerning the subject accrual process, baseline characteristics of enrolled subjects, degree of maternal depression symptoms and co-morbid conditions, and health status of the infants will be monitored on a weekly basis. Investigators will meet weekly with intervention providers and research assistants to discuss all active study participants.

Monitoring will pay particular attention to adverse events or events that have the potential to become adverse. Adverse events for this study will include suicide attempts, mandatory reports to child protective services for suspected abuse or neglect, new (i.e. previously unknown) domestic violence situations, complaints from study subjects, or any instance of breach of privacy or confidentiality. In addition, we will continually monitor for worsening depressive symptoms, and activate a crisis management plan as described in the IRB protocol (attached to the INSPIR application). Each time this

protocol is activated, we will analyze if the protocol functioned as expected; if it did not, that will also be considered an adverse event. Reporting of adverse events and unanticipated problems to the BUMC IRB will be done by the study team and occur in compliance with IRB reporting policies.

Dr. Silverstein will meet with the site co-investigators and Dr. Cabral (statistician) every two weeks to review data for the overall project. In case of an unanticipated problem or adverse event, co-investigators will notify Dr. Silverstein and Dr. Colin Sox (member of the Data Safety Monitoring Board). If central monitoring uncovers an unanticipated problem based on review of the aggregate data, then Dr. Silverstein will alert the site co-investigators of the issue, as well as the Boston University Medical Center IRB.

Please see the Appendix for details about our Data Safety Monitoring Board (DSMB).

10.3 Reporting Plans

The Principal Investigator at BMC/BU Medical Campus will report Unanticipated Problems, safety monitors' reports, and Adverse Events to the BMC/BU Medical Center IRB in accordance with IRB policies:

- Unanticipated Problems occurring at BMC/BU Medical Campus involving a fatal or life-threatening event will be reported to the IRB within 2 days of the investigator learning of the event.
- Unanticipated Problems occurring at BMC/BU Medical Campus not involving a fatal or life-threatening event will be reported to the IRB within 7 days of the investigator learning of the event.
- Reports from safety monitors with recommended changes will be reported to the IRB within 7 days of the investigator receiving the report.
- Adverse Events (including Serious Adverse Events) will be reported in summary at the time of continuing review, along with a statement that the pattern of adverse events, in total, does not suggest that the research places subjects or others at a greater risk of harm than was previously known.
- Reports from safety monitors with no recommended changes will be reported to the IRB at the time of continuing review.

The Principal Investigator will report Unanticipated Problems and Adverse Events to the BUMC IRB and to the study's Data Safety Monitoring Board (DSMB).

The DSMB will communicate its reports and recommendations on a quarterly basis after reviewing comprehensive project data. The DSMB will furnish a letter with its recommendations to Dr. Silverstein.

10.4 Stopping Rules

The study has no stopping rules.

11 Data Handling and Record Keeping

11.1 Confidentiality

All subjects will be assigned a unique study code. We will maintain subject confidentiality in the following ways:

1. The cross-walk that links study codes with identifying information will be kept in a secure network location and in a REDCap database. There will be 2 separate REDCap databases: one solely for identifying information and one solely for study data.
2. We will be using REDCap, a database intended for large research studies which can securely manage HIPAA-sensitive data.
3. All subjects will be made aware that no one from the clinical staff (aside from those directly involved in the study) will have access to any information.
4. All intervention sessions will take place in private areas.
5. Subjects will be given options to maintain confidentiality during contact. For example, if it is okay to leave a message and what specifically to say during the message from study staff to maintain confidentiality.

The study monitor or other authorized representatives of the sponsor may inspect all documents and records required to be maintained by the investigator, including but not limited to, medical records (office, clinic, or hospital) and pharmacy records for the subjects in this study. The clinical study site will permit access to such records.

11.2 Source Documents

The only source data/document needed for this study that is part of the subject’s standard of care treatment outside of the research is the EPDS screen. Responses will be entered by potential subjects either physically (on paper) or electronically (ie. on an iPad), depending on clinic resources. These results will later be entered into the subjects’ EHR by clinic staff.

All other study data will be obtained directly from subjects by study staff at study visits.

11.3 Case Report Forms

The study case report form (CRF) will be the primary data collection instrument for the study. All data requested on the CRF will be recorded. All missing data will be explained. If a space on the CRF is left blank because the procedure was not done or the question was not asked, “N/D” will be written. If the item is not applicable to the individual case, “N/A” will be written. All entries will be printed legibly in black ink. If any entry error has been made, to correct such an error, a single straight line will be drawn through the incorrect entry and the correct data will be entered above it. All such changes will be initialed and dated. There will be no erasures or white-out on CRFs. For clarification of illegible or uncertain entries, the clarification will be printed above the item, then initialed and dated.

Please see the Study Attachments in INSPIR for the following CRFs:

- Edinburgh Postnatal Depression Scale (EPDS)

- Suicide Screen
- MacArthur Competence Assessment Tool for Clinical Research (MacCAT-CR)
- Baseline Questionnaire
- Follow-up Questionnaires
- Collaborative Psychiatric Epidemiology Survey
- Child Behavior Checklist
- Parenting Stress Index Short Form
- Brief COPE
- Behavioral Activation for Depression Scale
- Beck Anxiety Index
- QIDS
- Verbal eligibility screen
- PSE Worksheet
- MINI (Mini International Neuropsychiatric Interview)

11.4 Study Records Retention

We will retain study records for at least 7 years after the completion of the study. We will retain documentation of informed consent for at least 3 years after completion of the study, after which we will shred or permanently delete such documentation.

12 Statistical Plan

12.1 Study Hypotheses

Given PSE's penetrance and acceptability in prior studies [21-23], we hypothesize that it's uptake will exceed 85% among women in this comparator; that it will provide sustained symptom relief; and that it will prevent the need for referring certain mothers (i.e. those with subsyndromal depression) to more formal care. By virtue of improved coping skills and behavioral activation, we hypothesize that PSE will make referrals to formal care more effective (for those who need it) than Engagement Interviewing alone. However, it is also important to assess the possibility that PSE may represent an unnecessary step that only serves to delay more definitive care and symptom relief. Our ultimate goal is to reduce disparities in access to mental health care for low-income mothers; and to improve outcomes for them and their children.

12.2 Sample Size Determination

Sample Size. For this project, our results would be used to build a case for intervention implementation within the PCMH. Because small differences in outcomes would fail to make this case effectively, we aim to detect a 33% relative reduction in symptomatic depressive episodes (defined as a bimonthly QIDS score ≥ 11) – which is both clinically important and consistent with previous work. All estimates assume 80% power and a two-sided alpha of 0.05.

1. **<u>Incident rate of moderate to severe symptomatic episodes</u>**. Using Poisson regression analysis, with 100 analyzable subjects in each study group and a Poisson rate of 1.2 symptomatic episodes per 6 follow-up assessments in one group (gleaned from data among a demographically similar population of peripartum women), we can detect a 33% reduction in the rate of episodes to 0.8 in the other group.
2. **<u>Depressive symptoms over time</u>**. In prior longitudinal data, we have observed the following mean QIDS scores for each follow-up month and assume these values for one group in the planned study: 8.2; 7.6; 7.5; 6.9; 7.2 (common SD, 5.9). We hypothesize that the following mean QIDS scores in the other group would be achievable and clinically significant: 7.2; 6.6; 5.5; 4.4; 4.2, resulting in a mean difference of 3 points at the final study time point in QIDS scores across study groups (note: a 3-point difference in QIDS scores, in the majority of cases, would move a subject across a clinical cutoff corresponding to mild, moderate or severe symptoms). Using a two-group repeated measures design analyzed by generalized estimating equations or mixed linear models, noting within-subject correlations of 0.7 for values separated in time by one month and 0.6 for those separated for two months or more in our longitudinal analysis, we can detect the above differences in the means between groups over time with 100 subjects per study group with 89% power for a correlation of 0.7 and 77% for correlation of 0.6.
3. **<u>HTE analysis</u>**. If we assume that equal numbers of women will be recruited pre- and postnatally, 100 subjects per arm will provide 86% power to detect differential effects of treatment between pregnant and postpartum women as extreme as a rate ratio of 0.38 in the prenatal stratum (rate in one group=0.45 vs. rate in the other=1.2) compared to a rate ratio of 0.96 in the postpartum stratum (rate in one group=1.15 vs. rate in the other=1.2).

Therefore, with an analyzable sample of 100 subjects per study group, we will be able to detect with adequate statistical power differences in incident rates of elevated QIDS scores and mean QIDS scores over time that are of clear clinical relevance. Based on our current data, we estimate that 20% of mothers with positive EPDS screens will refuse participation; 77% will meet full eligibility criteria; and we will experience a 15% attrition rate over the course of the study. Therefore, to ensure that we assess outcomes in 200 study subjects, we will approach 374 mothers with depressive symptoms and randomize 230. This sample size will be readily available from our two clinic sites.

12.3 Statistical Methods

Data Analysis Plan. We will report our results according to CONSORT guidelines. Because our aim is also for dissemination and implementation, we will analyze our data according to the RE-AIM (Reach, Effectiveness, Adoption, Implementation, Maintenance) framework, which addresses both internal and external validity.

1) **<u>Reach.</u>** We will record the number of mothers approached, screened, eligible, and refusing participation; compare demographics of those refusing to those participating; record subject attrition; and note all adverse events. We will compare baseline characteristics across comparators.

2) **<u>Effectiveness.</u>** We will conduct an <u>intention-to-treat analysis</u> to compare differences between comparators for all primary and secondary outcomes.

a) Main Effects. Our main effects analysis answers the question: **Which strategy is more effective at improving outcomes among a screened population of pregnant and postpartum women with depressive symptoms?**

- Patient Reported Outcomes: To assess depressive symptoms, we will employ Poisson or negative binomial regression techniques to compare incident rates of symptomatic episodes (QIDS ≥ 11) over 12 months of follow-up. We will use generalized estimating equations or mixed linear models to examine group-by-time effects on mean depressive symptom scores – as well as on mean scores of each of our other measures – as a function of intervention arm.
- Engagement and retention in care: Depending on the frequency of the observed outcomes, we will employ logistic or binomial regression techniques to compare the proportion of participants who meet the definition of engagement, retention, or receipt of evidence-based care. Among those engaging with care, we will employ Poisson or negative binomial regression techniques (depending on data dispersion) to compare number of mental health visits attended. We will compare treatment choices across groups. If data distribution assumptions are not met, we will consider nonparametric procedures or transformation of data. We will assess clustering by enrollment site and intervention practitioner, and if necessary use generalized mixed models to account for these effects. If randomization fails to produce balanced groups, we will add to our models potentially confounding variables, for which distributions are unequal across groups.

b) Heterogeneity of Treatment Effect (HTE). The primary goal of our HTE analysis is to determine whether there is differential impact across our comparators, according to timing of screening (pregnancy vs. postpartum). Our hypothesis-driven HTE analysis thus answers the question: **When is the optimal time to deploy each comparator?** We will also explore HTE according to whether our participants have experienced violent trauma.

- Timing of Screening. The most likely HTE scenario is that Engagement-Focused Care Coordination will compare less favorably during pregnancy, when women are averse to medication treatment, and apt to misinterpret a referral to outside treatment as a road leading for pharmacotherapy.
- Trauma. In our own studies, we've documented a possible differential impact of PSE according to trauma history; however, because these data were not generated in the context of a comparative effectiveness trial, we consider our HTE-by-trauma analysis to be exploratory.

For both HTE analyses, we will use Rothman's methodology, evaluating moderation with stratified analyses (according to categorical predictors), followed by formal interaction term testing in our models. We will analyze repeated symptom measures as time-dependent covariates, and test them for interaction relative to concurrent outcomes. Within each subgroup, we will present treatment effect estimates and 95% confidence intervals.

3) **Adoption.** We will collect data related to the characteristics of the prenatal and pediatric clinics that may influence adoption (including size of staff, number of families served), and assess through qualitative techniques the potential for adoption of the intervention in these clinical venues.

4) **Implementation**. This is the subject of a separate IRB application.

5) **Maintenance.** Within the purview of this study, we will be unable to assess maintenance on the institutional level; however, at the individual level, our follow-up period will span 12 months.

13 Ethics/Protection of Human Subjects

This study is to be conducted according to applicable US federal regulations and institutional policies (which are based in federal regulations, guidance, and ICH Good Clinical Practice guidelines).

This protocol and any amendments will be submitted to the Boston Medical Center and Boston University Medical Campus IRB, for formal approval of the study conduct. The decision of the IRB concerning the conduct of the study will be made in writing to the investigator. A copy of the initial IRB approval letter will be provided to the sponsor before commencement of this study.

All subjects for this study will be provided a consent form describing this study and providing sufficient information for subjects to make an informed decision about their participation in this study. The consent form will be submitted with the protocol for review and approval by the IRB. The consent of a subject, using the IRB-approved consent form, must be obtained before that subject is submitted to any study procedure. Consent will be documented as required by the IRB.

14 References

References

1. Siu, A.L., et al., *Screening for Depression in Adults: US Preventive Services Task Force Recommendation Statement.* JAMA, 2016. **315**(4): p. 380-7.
2. O'Connor, E., et al., *Primary Care Screening for and Treatment of Depression in Pregnant and Postpartum Women: Evidence Report and Systematic Review for the US Preventive Services Task Force.* JAMA, 2016. **315**(4): p. 388-406.
3. England, M.J. and L. Sim, *Depression in Parents, Parenting, and Children: Opportunities to Improve Identification, Treatment, and Prevention*. 2009, Institute of Medicine, National Academy Press: Washington, DC.
4. *Mental Health: Culture, Race, and Ethnicity: A Supplement to Mental Health: A Report of the Surgeon General*. 2001, United States Department of Health and Human Services, Public Health Service, Office of the Surgeon General: Rockville, MD.
5. Wang, P.S., P. Berglund, and R.C. Kessler, *Recent care of common mental disorders in the United States : prevalence and conformance with evidence-based recommendations.* J Gen Intern Med, 2000. **15**(5): p. 284-92.
6. Curran, G.M., et al., *Effectiveness-implementation hybrid designs: combining elements of clinical effectiveness and implementation research to enhance public health impact.* Med Care, 2012. **50**(3): p. 217-26.
7. Administration, U.S.D.o.H.a.H.S.-S.A.M.H.S. 2009 [cited 2009 August 31]; Available from: http://sbirt.samhsa.gov/about.htm.
8. Grote, N.K., et al., *A randomized controlled trial of culturally relevant, brief interpersonal psychotherapy for perinatal depression.* Psychiatr Serv, 2009. **60**(3): p. 313-21.
9. Grote, N.K., et al., *Engaging women who are depressed and economically disadvantaged in mental health treatment.* Soc Work, 2007. **52**(4): p. 295-308.
10. Huibers, M.J., et al., *The effectiveness of psychosocial interventions delivered by general practitioners.* Cochrane Database Syst Rev, 2003(2): p. CD003494.
11. Unutzer, J., et al., *Collaborative care management of late-life depression in the primary care setting: a randomized controlled trial.* Jama, 2002. **288**(22): p. 2836-45.

12. Pappas, G., et al., *Potentially avoidable hospitalizations: inequalities in rates between US socioeconomic groups.* Am J Public Health, 1997. **87**(5): p. 811-6.
13. Silverstein, M., et al., *Functional Interpretations of Sadness, Stress and Demoralization among an Urban Population of Low-Income Mothers.* Matern Child Health J, 2009.
17. Abidin, R., *Parenting Stress Index: a measure of the parent-child system*, in *Evaluating Stress: a book of resources*, C. Zalaqyuett and R. Wood, Editors. 1997, Scarecrow Press, Inc.: Lanam, MD. p. 277-291.
18. Heeringa, S.G., et al., *Sample designs and sampling methods for the Collaborative Psychiatric Epidemiology Studies (CPES).* Int J Methods Psychiatr Res, 2004. **13**(4): p. 221-40.
19. Katon, W., et al., *Collaborative management to achieve treatment guidelines. Impact on depression in primary care.* Jama, 1995. **273**(13): p. 1026-31.
20. Wells, K.B., et al., *Impact of disseminating quality improvement programs for depression in managed primary care: a randomized controlled trial.* Jama, 2000. **283**(2): p. 212-20.
21. Feinberg, E., et al., *Improving Maternal Mental Health After a Child's Diagnosis of Autism Spectrum Disorder: Results From a Randomized Clinical Trial.* JAMA Pediatr, 2014. **168**(1): p. 40-6.
22. Feinberg, E., et al., *Adaptation of problem-solving treatment for prevention of depression among low-income, culturally diverse mothers.* Fam Community Health, 2012. **35**(1): p. 57-67.
23. Silverstein, M., et al., *Problem-solving education to prevent depression among low-income mothers of preterm infants: a randomized controlled pilot trial.* Arch Womens Ment Health, 2011. **14**(4): p. 317-24.

15 Appendix

Schedule of Events (see below)
DSMB Charter (see below)

Protocol Appendix: Schedule of Events

- Pre-Randomization

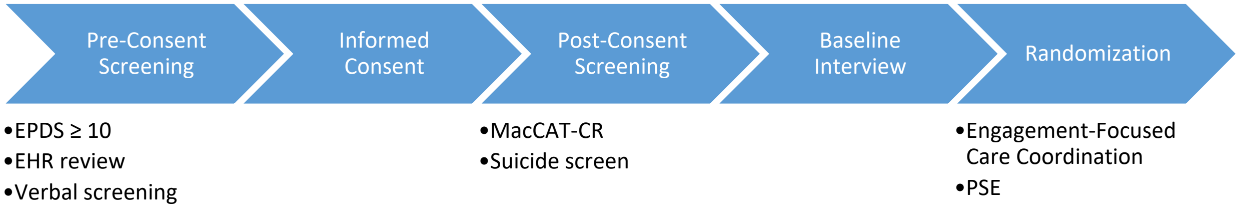

- Post-Randomization

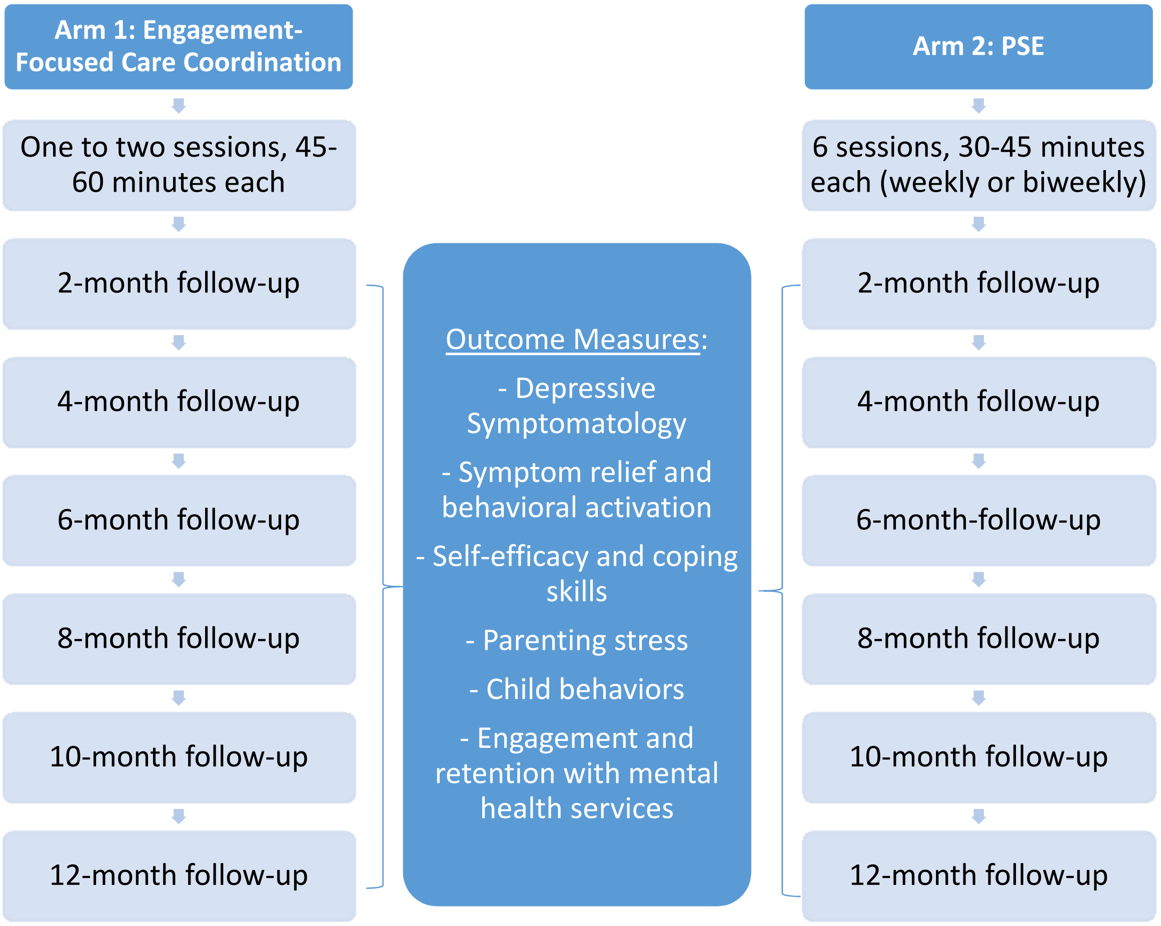

Protocol Appendix: DSMB Charter

A committee of three individuals (none of whom report to the PI or supervise the PI in any capacity) will serve as our Data Safety Monitoring Board:

- David Henderson, MD, is Professor of Psychiatry and Chair of the Department of Psychiatry at the Boston University School of Medicine/Boston Medical Center. He combines clinical mental health experience with a public health background.
- Colin Sox, MD, MS is Assistant Professor of Pediatrics at Boston University. His expertise is in conducting large scale safety studies that involve at-risk children. He is formally trained in epidemiology and biostatistics.
- Patricia Francisco, Patient Partner.

The committee will review comprehensive project data on a quarterly basis, including enrollment by site, follow-up data, study withdrawals, referrals to formal mental health services, and all adverse events (as defined in the protocol). The DSMB will determine if any of the above parameters, taken in aggregate, represent unanticipated problems. This quarterly standing meeting will be combined with expedited reporting to the DSMB of any unanticipated problems from the individual sites or from central monitoring by the overall PI. After each meeting, the DSMB will furnish a letter with its recommendations to Dr. Silverstein, who will send it to each site co-investigator. In the event that professional responsibilities change and a member of the DSMB has to report to Dr. Silverstein for other purposes (or vice-versa), we will identify another qualified DSMB member as a replacement.
