# Supplementary material for: Effect of Immediate Referral vs a Brief Problem-solving Intervention for Screen-Detected Peripartum Depression: A Randomized Clinical Trial
Source: JAMA Netw Open. 2023 May 12;6(5):e2313151. doi: 10.1001/jamanetworkopen.2023.13151 (PMC10182435; doi:10.1001/jamanetworkopen.2023.13151)
Supplement: Supplement 2. — eTable 1. Excluded Participants, by Recruitment Venue eTable 2. Depression and Anxiety Symptom Trajectories eTable 3. Incident Rates of Episodes of Clinically Significant Depression and Anxiety Symptoms, by Recruitment Venue eTable 4. Experience of Trauma on the Relationship Between Comparator and Outcomes [file jamanetwopen-e2313151-s002.pdf]

Supplementary Online Content

Elansary M, Kistin CJ, Antonio J, et al. Effect of immediate referral vs a brief problem-solving intervention for screen-detected peripartum depression: a randomized clinical trial. *JAMA Netw Open*. 2023;6(5):e2313151. doi:10.1001/jamanetworkopen.2023.13151

eTable 1. Excluded Participants, by Recruitment Venue
eTable 2. Depression and Anxiety Symptom Trajectories
eTable 3. Incident Rates of Episodes of Clinically Significant Depression and Anxiety Symptoms, by Recruitment Venue
eTable 4. Experience of Trauma on the Relationship Between Comparator and Outcomes

This supplementary material has been provided by the authors to give readers additional information about their work.

© 2023 Elansary M et al. *JAMA Network Open*.

eTable 1. Excluded Participants, by Recruitment Venue

**Reason**	**No. % Excluded, by Recruitment Venue**		
	**Prenatal Clinic n=259**	**Postpartum Unit n=23**	**Pediatric Primary Care n=58**
Not meeting inclusion criteria	112 (43)	4 (17)	20 (34)
Unreachable	57 (22)	12 (52)	19 (33)
Declined – Not interested in research	15 (6)	0 (0)	1 (2)
Declined- time considerations	75 (29)	7 (30)	18 (31)

© 2023 Elansary M et al. *JAMA Network Open*.

eTable 2. Depression and Anxiety Symptom Trajectories

**Model**	**Treatment-x-time interaction term for mean depression and anxiety scores**	
	**Mean Depression Scores**	**Mean Anxiety scores**
**Baseline symptom scores excluded**		
[a]Adjusted for baseline score	-0.34(-0.60, -0.08) p=0.009	-0.52 (-1.04, 0.00) p=0.05
Unadjusted	-0.41(-0.67, -0.14) p=0.003	-0.55 (-1.07, -0.02) p=0.04
**Baseline symptom scores included**		
Unadjusted	-0.29 (-0.51, -0.08) p=0.008	-0.24 (-0.67, 0.20) p=0.29

[a]Modeling strategy presented in Figure 2 of main manuscript

Generalized estimating equations were used to examine treatment-x-time effects on mean depressive and anxiety symptom scores. Problem Solving Education is the referent group. We estimated a primary model that excluded baseline symptoms in the set outcomes, but adjusted for them (modeling strategy presented in Figure 2). To ensure stability of results across plausible models, we also estimated an unadjusted model that included baseline symptoms in the set of outcomes; and an unadjusted model that excluded baseline symptoms in the set of outcomes. Statistically significant interaction terms indicate that trajectories differ, and all differences observed favor Engagement Focused Care Coordination.

© 2023 Elansary M et al. *JAMA Network Open*.

eTable 3. Incident Rates of Episodes of Clinically Significant Depression and Anxiety Symptoms, by Recruitment Venue

2A: Moderately Severe Depressive Symptom Episodes			
	**Engagement Focused Care Coordination (n=115)**	**Problem Solving Education (n=115)**	**aIRR (95% CI)**
Overall	2.2 (2.2)	2.2 (2.1)	0.95 (0.77, 1.17)
Prenatal Enrollment	1.8 (2.2)	1.9 (1.8)	0.85 (0.60, 1.22)
Postnatal Enrollment	2.4 (2.3)	2.5 (2.3)	1.01 (0.78, 1.30)
**2B: Severe Depressive Symptom Episodes**			
Overall	1.2 (1.6)	1.3 (1.7)	0.84 (0.63, 1.12)
Prenatal Enrollment	1.1 (1.6)	1.1 (1.5)	0.85 (0.54, 1.35)
Postnatal Enrollment	1.2 (1.7)	1.5 (1.9)	0.83 (0.58, 1.20)
**2C. Proportion of Participants with Moderately Severe Anxiety Symptom Episode**			
Overall	1.1 (1.8)	1.1 (1.6)	0.98 (0.69, 1.39)
Prenatal Enrollment	1.0 (1.8)	0.8 (1.5)	0.90 (0.50, 1.62)
Postnatal Enrollment	1.2 (1.8)	1.3 (1.7)	1.00 (0.66, 1.53)

The Engagement Focused Care Coordination and Problem Solving Education columns convey the mean number of follow-up symptomatic episodes (and SD) for each respective intervention comparator. The aIRR is the adjusted ratio between the two, adjusted for potential confounders and offset by the number of times each study participant contributed follow-up data. PSE is the referent group, and results are adjusted for baseline symptoms.

© 2023 Elansary M et al. *JAMA Network Open*.

eTable 4. Experience of Trauma on the Relationship Between Comparator and Outcomes

Model	Moderate depressive symptoms	Severe depressive symptoms	Anxiety symptoms
	P value for interaction term		
Trauma	0.64	0.88	0.29

© 2023 Elansary M et al. *JAMA Network Open*.
