# Supplementary material for: Effect of Immediate Referral vs a Brief Problem-solving Intervention for Screen-Detected Peripartum Depression: A Randomized Clinical Trial
Source: JAMA Netw Open. 2023 May 12;6(5):e2313151. doi: 10.1001/jamanetworkopen.2023.13151 (PMC10182435; doi:10.1001/jamanetworkopen.2023.13151)
Supplement: Supplement 3. — Data Sharing Statement [file jamanetwopen-e2313151-s003.pdf]

Data Sharing Statement

Elansary. Effect of Immediate Referral vs a Brief Problem-Solving Intervention for Screen-Detected Peripartum Depression. *JAMA Netw Open*. Published May 12, 2023. doi:10.1001/jamanetworkopen.2023.13151

Data
Data available: Yes
Data types: Deidentified participant data
How to access data: Researchers interested in accessing the deidentified public use dataset may submit a request in writing to the senior author.
When available: With publication

Supporting Documents
Document types: None

Additional Information
Who can access the data: Researchers whose proposed use of the data has been approved.
Types of analyses: Requests for data should specify proposed analyses. Scientific merit of the requests will be reviewed by the study team.
Mechanisms of data availability: Data will be made available without investigator support after approval of a proposal.
